# Impact of caring for patients with polyhandicap on institutional health care workers’ quality of life: a cross-sectional and longitudinal evaluation

**DOI:** 10.3389/fpubh.2024.1427289

**Published:** 2024-10-18

**Authors:** Marie-Christine Rousseau, Any Beltran, Ilyes Hamouda, Marie-Anastasie Aim, Agnès Felce, Katia Lind, Nafissa Khaldi, Houria El Ouazzani, Pascal Auquier, Thierry Billette de Villemeur, Karine Baumstarck

**Affiliations:** ^1^EA 3279, CEReSS - Research Centre on Health Services and Quality of Life, Aix Marseille University, Marseille, France; ^2^Hôpital San Salvadour, Assistance Publique Hôpitaux de Paris, Hyères, France; ^3^Department of Epidemiology and Health Economics, Assistance publique des hôpitaux de Marseille, Marseille, France; ^4^CRESCO, Paul Sabatier University, Université Toulouse III, Toulouse, France; ^5^Hôpital Marin d’Hendaye, Hendaye, Assistance Publique-Hôpitaux de Paris, Paris, France; ^6^Union Générale Caisse Assurance Maladie (UGECAM), Paris, France; ^7^Comité d’Études, d’Éducation et de Soins Auprès des Personnes Polyhandicapées, CESAP, Paris, France; ^8^Sorbonne Université, APHP.SU, Neuropédiatrie – Hôpital Trousseau – La Roche Guyon, Paris, France

**Keywords:** healthcare workers, longitudinal evaluation, polyhandicap, profound intellectual multiple disabilities, quality of life

## Abstract

**Background:**

Profound intellectual multiple disabilities or polyhandicap (PLH) is defined as a combination of profound mental retardation and serious motor deficits resulting in extreme dependence. Support for these patients is multidisciplinary, complex, and time-consuming. Thus, institutional health care workers (HCWs) face specific working conditions: frequent physical tasks, distressed families, and restricted feedback.

**Objectives:**

We aimed to identify determinants of quality of life (QoL) of HCWs and to study longitudinal evolution.

**Methods:**

The study used data from the French cohort EVAL-PLH. The participants were institutional HCWs of persons with PLH (age ≥ 3 years at the time of inclusion; age at onset of cerebral lesion <3 years old). Two populations were used: (1) cross sectional study: the sample 1 includes the HCWs assessed at T2 (2020–2021); (2) longitudinal study: the sample 2 includes the HCWs assessed at both T1 (2015–2016) and T2 (2020–2021). The data collected included: sociodemographics, health status, professional variables, and psycho-comportemental aspects. QoL was assessed using WHOQOL-BREF which provides 4 scores.

**Results:**

In comparison with French norms, the physical and social scores of QoL were significantly lower while the psychological score was significantly higher for (i) the 223 HCWs (participation rate 62%) assessed at T2 and (ii) the 61 HCWs assessed at T1 and T2. The main factors modulating QoL were age, marital status, self-perceived financial difficulties, personal chronic disease, anxiety-mood disorders, nature of coping strategies, and burnout.

**Conclusion:**

This study confirms the mixed (negative and positive) impact of caring persons with PLH on the institutional HCWs’ QOL. Main determinants of the HCW’s QOL were: older age, single status, perceived financial difficulties, altered health status, burn out and coping strategies.

**Clinical trial registration number**: NCT02400528.

## Introduction

Polyhandicap (PLH) is the consequence of a disorder affecting an immature brain, leading to profound intellectual impairment and serious motor deficits and resulting in extreme restrictions of autonomy and communication. Indeed, it represents one of the most severe chronic and complex conditions of disability and requires lifelong specialised care. Polyhandicap is close to the notion of Profound Intellectual and Multiple Disabilities (PIMD) that is used in other countries, and it does not systematically refer to a disorder affecting an immature brain (1). The term polyhandicap is used in several European and non-European countries, and recently, the Ithaca European Reference Network for congenital malformations and rare intellectual disabilities has agreed on the term PIMD/PLH. The prevalence of PLH is estimated to be approximately 1 per thousand (2–4), although robust data are lacking as they depend on the definition used.

While persons with PLH present varying severities of disorders and comorbidities they require, throughout their lives, both specific medical and technical care. To offer them a coherent, adapted, and integrative life they need preventive actions taking into account social and educational dimensions. The French health system allows persons with PLH to benefit from two main institutional care management modalities according to their specific needs in terms of health severity and medical and educational care. Specialised rehabilitation centres admit persons with PLH requiring a high level of medical and paramedical rehabilitation and the residential facilities admit persons requiring a lower level of medical care and high level of psychosocial education (5). Professional health care workers (HCWs) working in these institutions face specific working conditions that include frequent physical tasks due to the complete physical dependence of the persons, challenging personal histories of these persons and their distressed families, and restricted feedback and recognition of the care they provide due to the limitations of communication with the persons (6–10). In addition, residential facilities and specialised rehabilitation centres face recruitment difficulties and a high absenteeism rate, which means frequent staffing tensions (11, 12).

Few publications focus on the quality of life of HCWs working in specialised institutions for persons with PLH. The existing publications focus on residence care staff for persons with PIMD (10) or on professionals in the support of family living with PIMD persons (13), whose problematics are different and cannot be compared. Therefore, a better understanding of the experience of HCWs working in institutions for persons with PLH May assist health facility managers and care teams in taking appropriate targeted actions.

The French national cohort, the EVAL-PLH (EVALuation PoLyHandicap) study, was designed to identify the socioeconomic, environmental, and epidemiological determinants of the health status of persons with polyhandicap and their carers (parents and HCWs) (14). In a first study, using the data collected from the first assessment (2015), we already showed the considerable impact on the quality of life for institutional HCWs of both specialised rehabilitation centres and residential facilities dedicated to persons with polyhandicap (15). These preliminary results need to be confirmed on further assessments of HCWs participating in this cohort study. The present study aims (i) to provide the institutional HCWs’ quality of life second assessment (2020–2021) and comparison with French age-and sex-matched groups; (ii) to identify the potential determinants of institutional HCWs’ QoL; and (iii) to provide longitudinal analyses of changes over time (first and second assessment) in HCWs’ quality of life.

## Participants and method

### General organisation of the EVAL-PLH cohort

The French national EVAL-PLH cohort (clinical trial registration number NCT02400528) is a prospective cohort study. Data were collected at 2 points: T1) first assessment: during 2015 and 2016, and T2) second assessment: during 2020 and 2021. The protocol study was previously published (14).

Various French centres, spread over different territories, were participants: four specialised rehabilitation centres for inpatients needing heavy medical care for long durations (many days, months) through conventional hospitalisation stays and eight residential facilities for inpatients and outpatients needing less heavy medical care [the French Comité d’Études, d’Education et de Soins Auprès des Personnes Polyhandicapées Association (CESAP)]. The present study focused on the institutional HCWs of the 12 centres participating. All institutional HCWs of the included patients in the EVAL-PLH cohort were eligible.

In each centre, the steering committee who led the survey (consisting of physicians caring for persons with PLH, epidemiologists, and psychologists) held a meeting with all institutional HCWs to explain the objectives and modalities of the study.

All HCWs present in the structure were invited to participate. When HCWs who participated in the T1 assessment were not available for the T2 assessment, the reasons for their nonparticipation (leaving the institution or declining to participate) were systematically collected. This study follows the STROBE guidelines.

### Selection criteria

Eligible criteria of institutional HCWs were as follows: age above 18 years; being an institutional referent HCW of at least one participant who was included in the cohort of persons with PLH (a referent HCW is designed by the health care team for each patient; the referent HCW is the resource person who has to coordinate various issues for and about the person with PLH, such as management care, family contact, administrative, and social issues); and agreeing to participate. The exclusion criterion was refusal to participate. In this study, persons with polyhandicap were defined by: age at onset of cerebral lesion <3 years old and combination of motor deficiency (tetraparesia, hemiparesis, paraparesia, extra pyramidal syndrome, cerebellar syndrome, neuromuscular problems) and profound intellectual impairment (IQ < 40) associated with everyday life dependence (Functional Independence Measure [FIM] < 55), and restricted mobility (Gross Motor Function Classification System levels III–V). All persons with polyhandicap were aged ≥3 years at the time of inclusion.

### Populations

Two populations were used: (1) cross sectional study: the sample 1 includes the HCWs assessed at T2; (2) longitudinal study: the sample 2 includes the HCWs assessed at both T1 and T2.

### Data collection

A self-report booklet (with prepaid return envelope) was given to each referent institutional HCW volunteering to participate. No specification was given about where they had to fill out the booklet (at the hospital or not). The booklet included the following data. (1) Sociodemographics: sex, age, marital status, children, educational level, self-perceived financial situation, and notion of a disabled person living at home. (2) Health: personal chronic disease(s), hospitalisation episode during the last year, and receiving antidepressant or anxiolytic drugs. (3) Professional characteristics: job categories: technical (nurses and specialised paramedical including physiatrists, psychometricians, and ergotherapists) vs. not technical (nurse aids and educators), work schedule (full-time, part-time), years of experience in care for persons with PLH, years of experience in the present structure, and nature of the structure (specialised rehabilitation centre, residential facility). (4) Job environment and satisfaction: safe handling training programmes provided by the structure (Yes/No), reported intention and reasons to quit the present institution (Yes/No), actively searching for another place (Yes/No) and satisfaction with the medical information provided about the person with PLH they care for (Yes/No). (5) Psycho comportemental characteristics: i) Anxiety-mood disorders assessed using a Likert scale ranging from 1 (absence) to 10 (very significant); ii) Occupational burnout assessed using the Maslach Burnout Inventory (MBI) recognised as a valid and reliable tool for occupational burnout syndrome assessment. The MBI provides a global score. From the global score, three levels of burn out were defined: high, moderate, and low (16); iii) Coping assessed using the Brief Coping Orientation to Problems Experienced Scale (Brief-COPE) (17) exploring four dimensions that include social support, problem solving, avoidance, and positive thinking (18). Scores ranged from 0 to 100. High scores reflect a high tendency to implement the corresponding coping strategies.

Quality of life (QoL) was assessed using the World Health Organisation Quality of Life (WHOQOL-BREF) questionnaire, a generic questionnaire used worldwide describing four domains: physical health, psychological health, social relationships, and environment. All scores ranged between 0 and 100. Higher scores indicated a better quality of life. French norms are available (16,392 healthy individuals) for three domains (physical, psychological and social) (19).

### Ethics

All methods were carried out in accordance with relevant French guidelines and French regulations. The research was approved by the French ethics committee (name: Comité de Protection des Personnes Sud Méditerranée V; postal address: CHU·HOPITAL DE CIMIEZ, Nice, France; website^1^; approbation date: 20/10/2014; reference number: 2014-A00953-44; check-in number: 14.041). The research was performed in accordance with the Declaration of Helsinki. A written consent form was collected for each participant.

### Statistics

The quantitative data are expressed as the means and standard deviations (SD) or the medians and interquartile ranges, and the qualitative data are expressed as numbers and percentages. The WHOQOL-BREF, the MBI, and the Brief-COPE were scored using the rules provided by their respective developers. Scores of WHOQOL-BREF of the institutional HCWs were compared with the French age- (six classes) and sex- (females/males) matched samples using paired *t* tests. Comparisons of the 4 QoL scores were performed using Student’s t tests or ANOVA tests for the following subgroups: sex (men vs. women), marital status (single vs. not single), having children (no vs. yes), educational level (< vs. > = 12 years), self-perceived financial status (not difficult vs. difficult), living with a person with disability (no vs. yes), chronic disease[s] (no vs. yes), job categories (technical vs. not technical care), work schedule (full vs. part-time), and nature of the structure (specialised rehabilitation centres vs. residential facility). Associations between the QoL scores and the continuous variables (age, years of care experience with persons with PLH, years of experience in the present centre, coping scores, and burn-out total score) were analysed using Pearson’s correlations. Multivariate analyses using multiple linear regressions were performed to identify the variables linked to the QoL scores. In the models, each QoL dimension score was considered to be a separate dependent variable. The independent variables relevant to the models were selected from the univariate analysis, based on a threshold *p* value of less than or equal to 0.05. The final models produced standardised beta coefficients, which represent a change in the standard deviation (SD) of the dependent variable (QoL score) resulting from a change of one SD in the various independent variables. Independent variables with higher standardised beta coefficients were those with a greater relative effect on QoL. All tests were two-sided. The threshold for statistical significance was set at *p* < 0.05. The statistics were analysed with SPSS software (IBM SPSS PASW Statistics Inc., Chicago, IL, United States).

## Results

**Figure 1:**
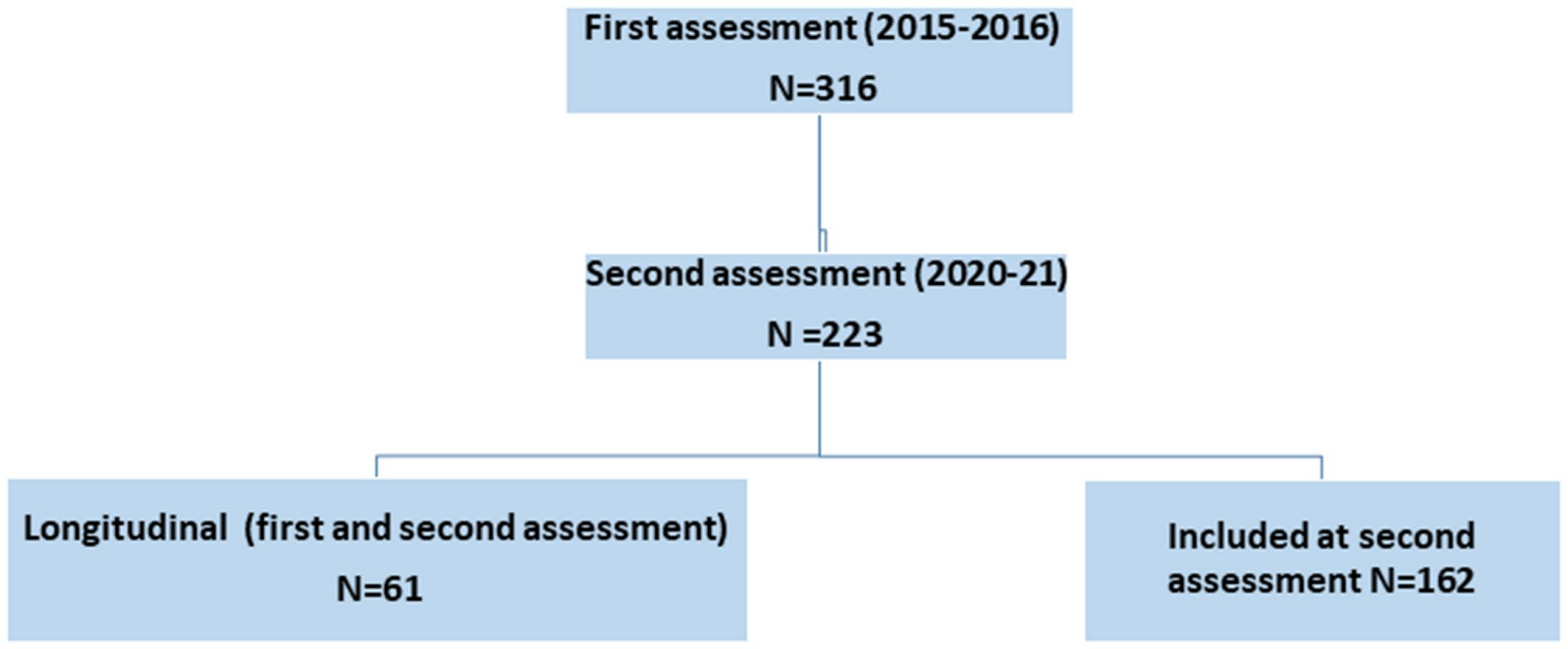
Flow chart.

From a total of 363 questionnaires (with prepaid return envelope) proposed to referent institutional HCWs of the 12 centres, between March 2020 and December 2021, 223 participants (sample 1) participated (response rate 62%). The 140 nonparticipants did not differ from the participants in terms of age and sex. Finally 61 participants (sample 2) at T1 returned their questionnaire at T2. For details see flow chart provided in Figure 1.

### General characteristics

#### HCWs assessed in 2020–2021: cross sectional analysis (sample 1)

The 223 participants were between the ages of 21 and 61 years old, and 182 (81.6%) were women. Almost 40 percent of the institutional HCWs reported self-perceived financial difficulties. More than 6 % reported living with a disabled person. Forty percent of the respondents reported at least one personal chronic disease (musculo skeletal disorders (67), asthma (12), cardiovascular disorders (6), mellitus diabetes (5), and other disorders (14)), 10.5 percent reported a hospitalisation episode during the last year, and almost 20 percent received antidepressant/anxiolytic drugs. Most of the institutional HCWs, 82.5 percent, were classified in the nontechnical care category (nurse aids, educators) and 17.5 percent were classified in the technical care category (nurses, physiatrists, psychomotricians, ergotherapists). The mean (SD) duration of experience in PLH care was 12 (9) years and the mean (SD) experience in the present structure was 10 (9) years. Sixty-seven percent of HCWs worked in specialised rehabilitation centres and one-third worked in residential facilities. Thirty-four percent of institutional HCWs benefited from safe handling training programmes provided by their institution, and 21% of institutional HCWs reported intention to quit the structure, reasons given by HCW to quit the institution were (workload (21), feeling of weariness (12), health problems (10) and other reasons 18)). Twelve percent of the HCWs actively searched for another job place. Sixty percent of HCWs reported being rather satisfied with the medical information provided about the person with PLH they care for. Thirty-eight percent of the institutional HCWs reported a high burnout level according to the MBI. The coping strategies that were based on avoidance and positive thinking were the least used, and the strategies based on problem solving and social support were the most-used. For details see Table 1.

**Table 1:** Characteristics of the 223 health care workers for T2 assessment (sample 1).

		Mean (SD); N (%)	MD; N (%)
**Sociodemographics**			
Women		182 (81.6)	0
Age (years)		42 (11)	4 (1.8)
Marital status	Not single	70 (32.4)	7 (3.1)
	Single	146 (67.6)	
Having children	No	61 (27.7)	3 (1.3)
	Yes	159 (72.3)	
Educational level	<12 years	75 (34.7)	7 (3.1)
	≥12 years	141 (65.3)	
Self-perceived financial status	Not difficult	133 (62.1)	9 (4)
	Difficult	81 (37.9)	
Disabled person living home	No	206 (93.6)	3 (1.3)
	Yes	14 (6.4)	
**Personal health**			
Personal chronic disease(s)	No	133 (61)	5 (2.2)
	Yes	85 (39)	
Hospitalisation episode during the last year	No	195 (89.4)	5 (2.2)
	Yes	23 (10.6)	
Antidepressants anxiolytics drugs	No	177 (81.2)	5 (2.2)
	Yes	41 (18.8)	
**Professional characteristics**			
Job categories	Nurses & specialised paramedical	38 (17.4)	4 (1.8)
	Nurse aids	154 (70.3)	
	Educators	27 (12.3)	
Job categories classes*	Technical care	38 (17.4)	4 (1.8)
	Nontechnical care	181 (82.6)	
Work schedule	Full time	184 (83.3)	2 (0.9)
	Part time	37 (16.7)	
Experience in polyhandicap care (years)		12 (9)	5 (2.2)
Experience in the present structure (years)		10 (9)	7 (3.1)
Nature of structure	Specialised rehabilitation centre	150 (67.3)	0
	Residential facility	73 (32.7)	
**Job environment and satisfaction**			
Safe handling training programmes	No	142 (65.5)	6 (2.7)
	Yes	75 (34.5)	
Intention to quit the structure	No	174 (79)	3 (1.3)
	Yes	46 (20.6)	
Reasons to quit	Workload	21	0
	Health problems	10	
	Weariness	12	
	Other reasons	18	
Actively searching for another job place	No	195 (87.4)	0
	Yes	28 (12.6)	
Satisfaction with the medical information provided about the person with PLH	Rather satisfied	133 (60.7)	4 (1.8)
	Rather not satisfied	86 (39.3)	
**Psycho-comportemental characteristics**			
Anxiety-mood score (1–10)**		5.56 (2.7)	6 (2.7)
Burnout (Maslach Burnout Inventory)	Total score***	-11 (14.3)	15 (6.7)
	Low level	53 (25.5)	
	Moderate level	76 (36.5)	
	High level	79 (38)	
Coping strategies (BriefCope)****	Social support	42.3 (21)	14 (6.2)
	Problem solvings	57.6 (23)	16 (7.17)
	Avoidance	26 (12)	17 (7.6)
	Positive thinking	53 (18.5)	17 (7.6)

*Job categories: technical (nurses, physiatrists, psychomotricians, ergotherapists), nontechnical (nurse aids, educators). **1 absence to 10 higher disorder. ***the higher the score the higher burnout level. ****scores ranging 0 to 100, the higher the score the higher coping strategy is used. SD, standard deviation; N (%), number (percent); MD, missing data.

#### HCWs assessed 2 times (sample 2)

The main characteristics of the 61 participants in the longitudinal evaluation are presented in Table 2.

### Quality of life

#### HCWs assessed in 2020–2021: cross sectional analysis (sample 1)

**Figure 2:**
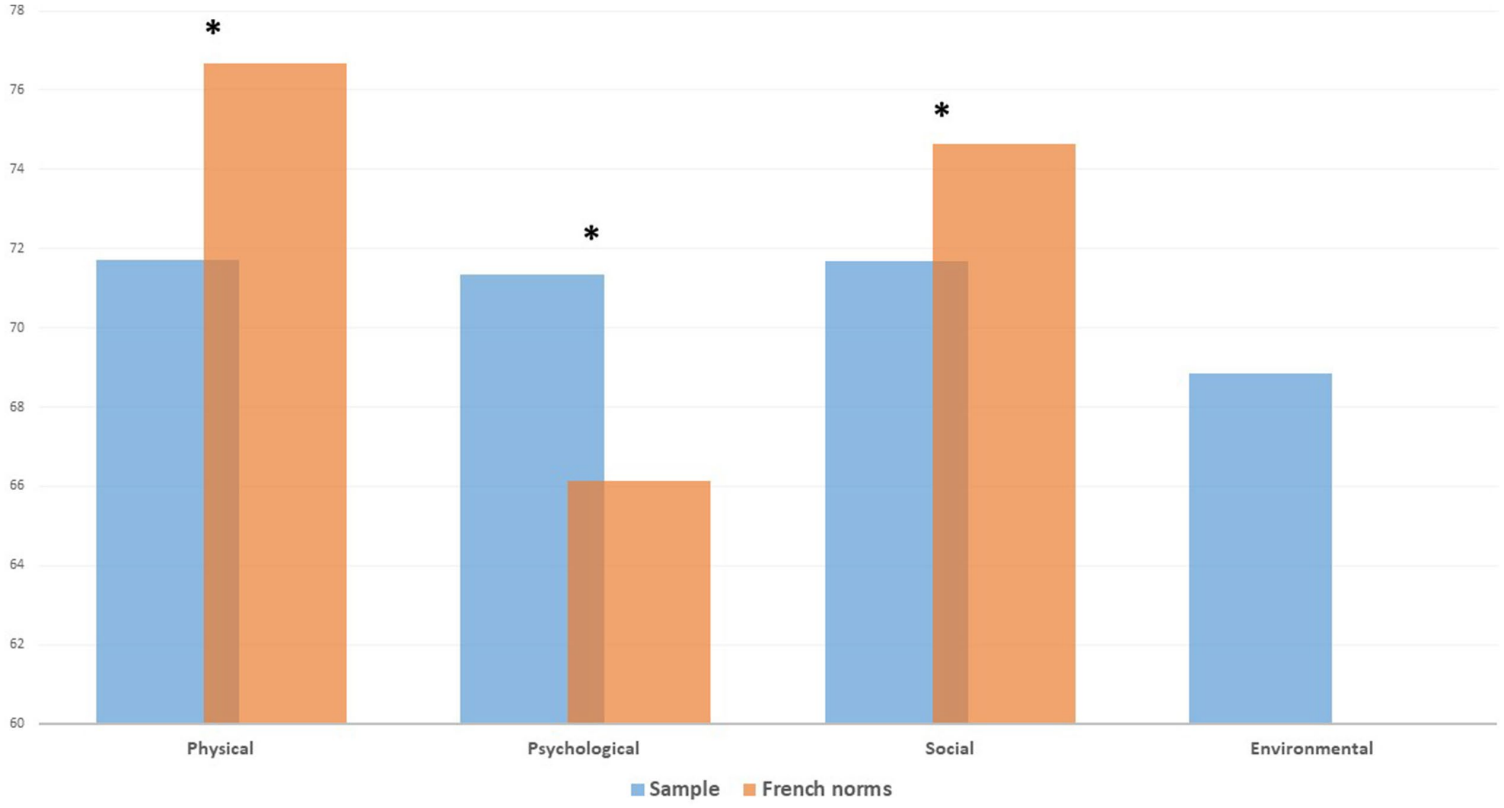
Institutional health care workers’ QoL for T2 assessment (sample 1).

Of the three dimensions of the WHOQOL-BREF for which French norms are available, compared with French norms (age-and sex-matched individuals), the HCWs presented significantly lower scores in the physical and social dimensions, and higher scores in the psychological dimension (see Figure 2).

#### HCWs assessed on the 2 times (sample 2)

**Figure 3:**
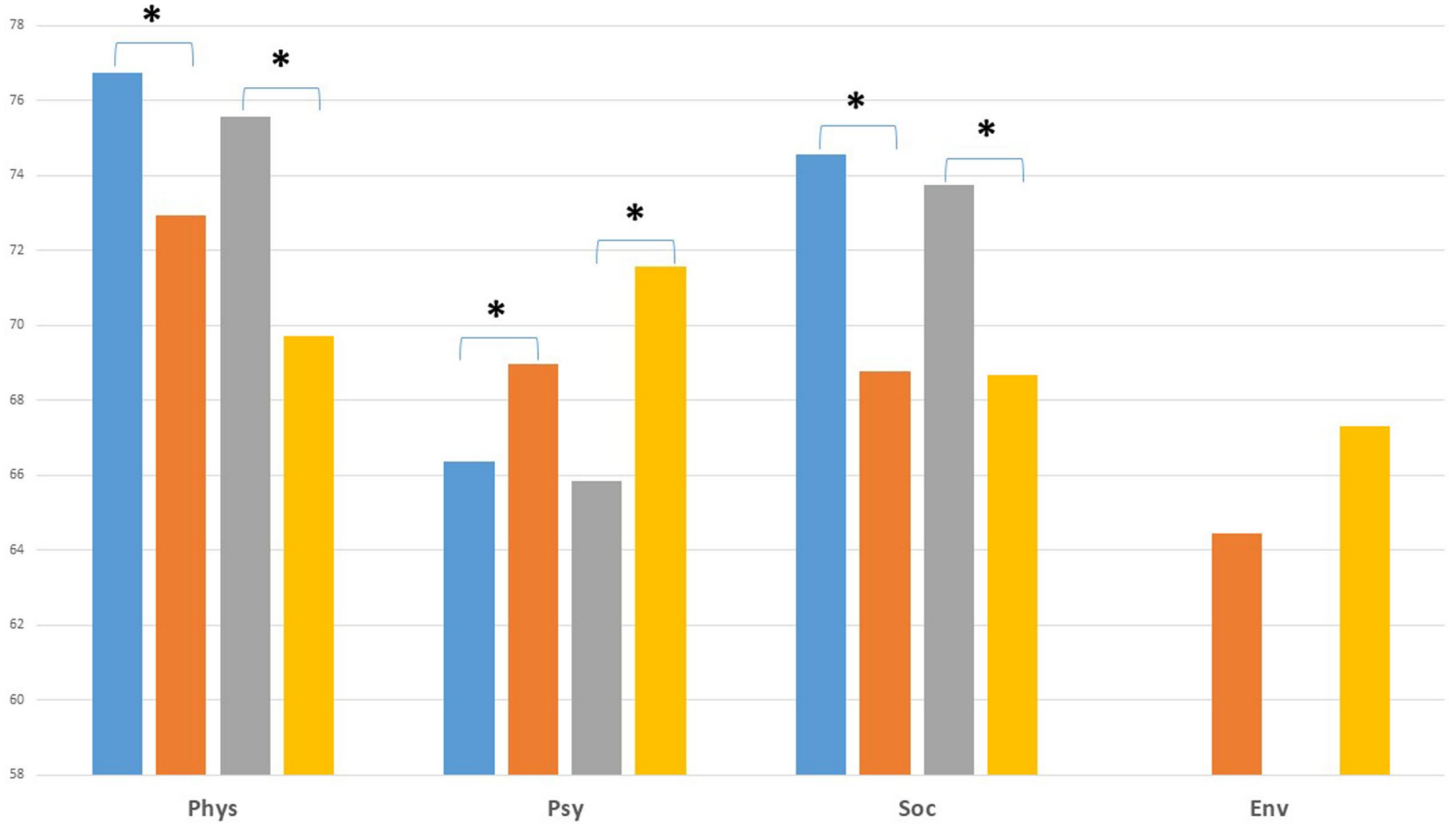
Institutional health care workers’ QoL for T1-T2 assessment (sample 2).

Longitudinal evaluation shows an accentuated trend in quality of life profiles, with lower scores at the T2 assessment (2020–2021) for the physical and social dimensions and higher scores for the psychological dimension compared to the T1 assessment (2015–2016). At T2, the HCWs reported significantly lower scores for the physical and social dimensions and higher scores for the psychological dimension in comparison with age-and sex-matched healthy individuals (see Figure 3).

### Factors modulating the QoL

The analysis of the factors modulating the QoL of the institutional HCWs was provided for the 223 HCWs assessed at the T2 assessment. The univariate analysis results are provided in Table 3.

**Table 2:** Characteristics of the 61 health care workers for T1-T2 assessment (sample 2).

		Mean (SD); N (%)	
		T1	T2
**Sociodemographics**			
Women		46 (75.4)	46 (75.4)
Age (years)		42.1 (8.7)	47.3 (8.6)
Marital status	Not single	37 (60.7)	41 (67.2)
	Single	23 (37.7)	18 (29.5)
Having children	No	18 (29.5)	10 (16.4)
	Yes	42 (68.9)	50 (82.0)
Educational level	<12 years	28 (45.9)	26 (42.6)
	≥12 years	31 (50.8)	34 (55.7)
Self-perceived financial status	Not difficult	3 (4.9)	36 (59.0)
	Difficult	57 (93.4)	23 (37.7)
Job categories classes*	Technical care	6 (9.8)	8 (13.1)
	Nontechnical care	46 (75.4)	51 (83.6)
Work schedule	Full time	48 (78.7)	46 (75.4)
	Part time	12 (19.7)	14 (23.0)
Experience in polyhandicap care (years)		12.6 (8.6)	16.9 (8.5)
Nature of structure	Specialised rehabilitation centre	50 (82.0)	50 (82.0)
	Residential facility	11 (18.0)	11 (18.0)
**Psycho-comportemental characteristics**			
Anxiety-mood score (1–10)**		4.8 (2.3)	5.2 (2.8)
Burnout (Malash Burnout Inventory)	Total score***	−12.9 (16.7)	−10.2 (15.8)
	Low level	19 (31.1)	15 (24.6)
	Moderate level	19 (31.1)	18 (29.5)
	High level	21 (34.4)	26 (42.6)
Coping strategies (BriefCope)****	Social support	41.6 (17)	41 (21.2)
	Problem solvings	61.2(20.2)	55 (23.5)
	Avoidance	28 (8.9)	29 (11.9)
	Positive thinking	57.5 (18.9)	55.8 (16.3)

*Job categories: technical (nurses, physiatrists, psychomotricians, ergotherapists), nontechnical (nurse aids, educators). **1 absence to 10 higher disorder. ***the higher the score the higher burnout level. ****scores ranging 0 to 100, the higher the score the higher coping strategy is used. SD, standard deviation; N (%), number (percent).

After adjustment (see Table 4), the factors modulating the QoL were as follows: (1) Alteration of the physical QoL dimension: being older (*b* = −0.157; *p* = 0.02), having a chronic disease (*b* = −0.280, *p* = <0.001), reporting a higher anxiety-mood level (*b* = −0.376, *p* < 0.001), not using problem solving as coping strategy (*b* = 0.158; *p* = 0.003) and having a higher burnout level (*b* = −0.141, *p* = 0.025). (2) Alteration of the psychological QoL dimension: reporting self-perceived financial difficulties (*b* = −0.122, *p* = 0.039), having a chronic disease (*b* = −0.119, *p* = <0.046), reporting a higher anxiety-mood level (*b* = −0.291, *p* < 0.001), and using avoidance as a coping strategy (*b* = −0.289; *p* < 0.001). (3) Alteration of the social QoL dimension: being older (*b* = −0.232; *p* = 0.008), single marital status (*b* = 168, *p* = 0.014), reporting a higher anxiety-mood level (*b* = −0.156, *p* = 0.044), and using avoidance as a coping strategy (*b* = −0.260; *p* < 0.001). (4) Alteration of the environmental QoL dimension: reporting self perceived financial difficulties (*b* = −0.362, *p* < 0.001), higher anxiety-mood disorders (*b* = −0.241, *p* < 0.001) and using avoidance as a coping strategy (*b* = −0.199; *p* = 0.002). Gender, experience in the present structure and positive thinking as a coping strategy were not associated with the QoL scores.

**Table 3:** Relationships between quality of life scores and characteristics of the223 health care workers (univariate analysis).

Sociodemographics			Physical	Psychological	Social	Environmental
Gender		Women	72 (15.3)	70.3 (13.4)	70.7 (15.7)	68.1 (12.3)
		Men	70 (19.3)	76 (13.3)	76 (14.3)	72.4 (13.7)
		*p*-value	0.703	**0.018**	0.052	0.050
Age (years)		R	−0.183	0.044	−0.176	0.051
		*p*-value	**0.0007**	0.518	**0.010**	0.457
Marital status		Single	71 (15.4)	69.7 (14.2)	67.6 (16.7)	68.1 (13)
		Not single	72.2 (15.6)	72.2 (13.2)	73.6 (15)	69.4 (12.4)
		*p*-value	0.735	0.212	**0.009**	0.482
Having children		No	72.8 (17)	68.8 (16)	72.6 (16.8)	67.8 (14)
		Yes	71.3 (16)	72.3 (12.5)	71.3 (15.1)	69.3 (12.2)
		*p*-value	0.524	0.138	0.601	0.454
Educational level		<12 years	70.3 (15)	70.4 (15))	70 (18.4)	68.1 (13.2)
		≥12 years	72.5 (16.4)	71.8 (13	72.6 (14)	69.3 (12.5)
		*p*-value	0.343	0.465	0.294	0.507
Self-perceived financial status		Not difficult	74.1 (14.4)	73.7 (12)	72.4 (14.6)	73.2 (10.5)
		Difficult	67.6 (18)	66.9 (14.6)	70 (17)	61.4 (12.6)
		*p*-value	**0.006**	**<0.001**	0.290	**<0.001**
Disabled person living home		No	72 (16)	71.5 (13)	72.2 (15)	69.1 (12.7)
		Yes	67.3 (16)	68.4 (20)	64.3 (22)	64.5 (12)
		*p*-value	0.295	0.581	0.205	0.190
**2. Personal health**						
Personal chronic disease(s)		No	77.7 (12.3)	73.8 (12.3)	73.7 (14.2)	70 (12)
		Yes	62.4 (16.7)	67.3 (14)	68.5 (17)	67 (13.2)
		*p*-value	**<0.001**	**<0.001**	**0.024**	0.092
Hospitalisation during the last year		No	71.6 (16.2)	71.1 (13.5)	71.6 (15.6)	68.9 (12.5)
		Yes	71.9 (15)	72.4 (13)	73.3 (15.7)	68 (13)
		*p*-value	0.942	0.678	0.832	0.771
**3. Job characteristics**						
Job categories classes*		Technical care	75.4 (15.8)	73.6 (12)	71.6 (13.6)	72.3 (10.7)
		Nontechnical care	71 (16)	70.8 (13.8)	71.6 (16)	68.2 (13)
		*p*-value	0.125	0.254	0.986	0.074
Work schedule		Full time	72.3 (16.2)	70.8 (13.5)	71.6 (15)	69 (12.6)
		Part time	68.8 (15.2)	74 (13.4)	72.1 (18.3)	68.2 (13.1)
		*p*-value	0.230	0.192	0.852	0.723
Experience in polyhandicap care (years)		R	−0.131	0.044	−0.066	0.067
		*p*-value	0.056	0.521	0.345	0.332
Experience in the present structure (years)		R	−0.178	0.005	−0.162	0.011
		*p*-value	**0.010**	0.938	**0.019**	0.872
Nature of structure		Specialised rehabilitation centre	72.7 (16.5)	71.6 (14.2)	72.5 (15.7)	68.9 (13.6)
		Residential facility	69.8 (15)	71 (12)	70 (15.1)	68.8 (10.5)
		*p*-value	0.222	0.783	0.288	0.943
**4. Psycho-comportemental characteristics**						
Anxiety-mood score (1–10)**		R	−0.536	−0.496	−0.261	−0.343
		*p*-value	**<0.001**	**<0.001**	**<0.001**	**<0.001**
Coping***	Social support	R	0.030	−0.024	0.006	−0.023
		*p*-value	0.663	0.734	0.937	0.737
	Problem solving	R	0.144	0.146	0.088	0.149
		*p*-value	**0.039**	**0.036**	0.209	**0.033**
	Avoidance	R	−0.256	−0.426	−0.300	−0.295
		*p*-value	**<0.001**	**<0.001**	**<0.001**	**<0.001**
	Positive thinking	R	0.132	0.247	0.131	0.172
		*p*-value	0.059	**<0.001**	0.063	**0.014**
Burnout**** Total score		R	−0.439	−0.405	−0.211	−0.264
		*p*-value	**<0.001**	**<0.001**	**0.002**	**<0.001**

*Job categories: technical (nurses, physiatrists, psychomotricians, ergotherapists), nontechnical (nurse aids, educators). **1 absence to 10 higher disorder. ****the higher the score the higher burnout level. ***scores ranging 0 to 100, the higher the score the higher coping strategy is used. Coefficient estimate: coefficient of regression slope from linear model. Bold values: *p*-value < 0.05.

**Table 4:** Relationships between quality-of-life scores and characteristics of the 223 health care workers (multifactorial analysis).

	Physical		Psychological		Social		Environmental	
	*β*	*p*-value	*β*	*p*-value	*β*	*p*-value	*β*	*p*-value
Gender (0 Men, 1 Women)	−	−	−0.115	0.051	−	−	−	−
Age (years)	−0. 157	**0.021**	−	−	−0.232	**0.008**	−	−
Marital status (0 single, 1 not single)	−	−	−	−	0.168	**0.014**	−	−
Self-perceived financial status (0 Not difficult, 1 Difficult)	−0.101	0.060	−0.122	**0.039**	−	−	−0.362	**< 0.001**
Personal chronic disease(s) (0 no, 1 yes)	−0.280	**< 0.001**	−0.119	**0.046**	0.034	0.647	−	−
Experience in the present structure (years)	−0.060	0.350	−	−	−0.056	0.494	−	−
Anxiety-mood score	−0.376	**< 0.001**	−0.291	**< 0.001**	−0.156	**0.044**	−0.241	**0.001**
Problem solving	0.158	**0.003**	0.098	0.4141	−	−	0.118	0.094
Avoidance	−0.076	0.173	−0.289	**< 0.001**	−0.260	**< 0.001**	−0.199	**0.002**
Positive thinking	−	−	0.091	0.192	−	−	0.018	0.801
Burnout Total score	−0.141	**0.025**	−0.066	0.344	−0.054	0.494	0.024	0.746

(−) Not include factor in a multifactorial analysis model. β, Standardised coefficient from multifactorial linear model. Bold values: *p*-value < 0.05.

## Discussion

Our study has several main findings with potential practical and research implications. First, our results confirm those of the first evaluation (2015–2016), with a significant impact on QoL compared to age and sex-matched French general populations. When regarding QoL domains, comparison with the general population QoL confirms on both cross sectional and longitudinal evaluations, the same contrasted pattern with a marked negative impact on the physical and social domains and on the opposite higher psychological quality of life of institutional HCWs.

A variety of factors explain the deterioration in the physical QoL of institutional HCWs; polyhandicapped persons are totally dependent, and these professionals sometimes struggle with physical demands such as repetitive lifts and handling from bed to sitting devices, for toileting and dressing. Nearly all of the dedicated institutions are actually equipped with transfer aid devices and at least 1/1 HCW/persons with a PLH ratio; however our results show that all these measures are still not sufficient in preserving their physical QoL. In addition, only a third of the institutional HCWs benefited from regular safe handling training programmes provided by their institution, which probably explains the high (30%) proportion of musculoskeletal disorders experienced by the HCWs.

These professionals face a population with complex health conditions and are constantly confronted with psychologically difficult situations such as certain symptoms (abnormal movements, screaming, crying), and managing relations with families (8). In addition, the mortality rate of persons with PLH is high (20% in a 5 years period; Hamouda unpublished data); thus providing care for such persons May entail a particular form of palliative care that extends over several years (20).

Nevertheless, they surprisingly have a higher psychological quality of life than the general population, and this trend continues to evolve over time. In fact, they play an essential role that encompasses the care of persons with PLH; Petry et al. previously showed that the support offered by institutional HCWs May influence the QoL of persons with PIMD (21, 22). The opportunity for institutional HCWs to have a positive influence on the wellbeing of the persons they care for, in a similar way to what has been observed among family carers of children with developmental disabilities (23, 24), contributes to strengthening their sense of meaning at work and empowerment. Similarly to what was observed for caregivers of persons with intellectual disability, institutional HCWs have a rather long experience of with polyhandicapped persons (mean 12 years) (7) with no influence on their QoL. These results emphasise the positive aspects of this profession: caring for persons with PLH May be considered a meaningful job, contributing to satisfactory self-esteem and psychological well-being. Although this finding needs to be tempered as the consumption of antidepressants or anxiolytics by HCWs of our study is almost twice as high (19%) as that of the French general population [antidepressants (6%) and anxiolytics (10%)] (25).

The negative social impact on QoL raises questions: working near people with very little communication ability who are unable to express their approval or disapproval, their feelings or their contentment, May generate a feeling of frustration for the institutional HCW. PLH is not a curable clinical condition implying that institutional HCWs renounce to the medical model of healing. A French study looking at the geographical location of care facilities for people with severe disabilities, revealed a somewhat segregative practice, showing a higher concentration of institutions for these people in the most isolated and least populated regions (CEDIAS, 2010). In fact, persons with PLH are on the opposite of what the modern individual imagines as a situation of social well-being, their dysmorphic appearance with major scoliosis, limb deformations, and sitting devices May be hard to assume (26). Even though professionals HCWs are committed to serving persons with PLH, which gives them a particular place in our society, it is probably difficult for them to share their particular professional experience with their social circle.

Several sociodemographic characteristics, including female gender, single status and perceived financial difficulties were identified as negative QoL determinants. The health status (chronic disease, anxiety, or mood disorders) of the institutional HCWs was directly related to their self-reported QoL. The HCWs with longer experience in the same institution experienced more deteriorated QoL. In addition, up to 20 % planned to quit their actual institution, mainly due to workload, feelings of weariness and health problems. This emphasises the need to create an attractive and supportive work environment that retains health professionals. Health awareness initiatives such as preventive health checkups should be proposed to prevent and diagnose health problems such as musculoskeletal problems and chronic diseases.

Psycho-comportemental characteristics strongly affect institutional HCW’s QoL. The nature of the coping strategies used plays a consequential role in the institutional HCW’s QoL modulation: the association between using avoidance as a coping strategy and worse individual outcomes has been described previously in various contexts, such as cancer (27) and severe mental illnesses (28). In contrast, a positive-thinking strategy arose as a protective strategy for QoL. Interventions based on problem solving or positive thinking have revealed predictors of satisfactory wellbeing in institutional HCWs, although targeted psychological interventions based on psychoeducation and cognitive behavioural therapy May be offered to these professionals.

Both the first and second assessments showed an important proportion of individuals with a high level of stress and burnout (15), which is probably one of the causes of the important absenteeism (average (9–11%)) (12) in these institutions. Kozak et al. previously showed that nurses working with people with intellectual disabilities who received more feedback from supervisors and colleagues had a reduced risk of burnout and managers should be aware that adequate information, feedback and acknowledgment on work carried out can be a resource to limit HCW’s burn-out (29). This result should alert us, and future studies should explore burn out and its determinants more closely.

### Strengths and limitations

One of the limitations of our study was the representativeness of our sample: the specialised rehabilitation centres allowed a satisfactory representation (70%) of the French health care system, although the representativeness of residential facilities, all belonging to a single association (CESAP) should be improved.

However, this study provides for the first time a longitudinal and dynamic perspective of the institutional HCW’s QoL confirming the negative (although mixed) impact of caring for persons with PLH. These findings have implications as several QoL factors are modifiable. Coping strategies May be improved and regular safe handling training programmes for HCWs should be implemented by institutions or health agencies. Better feedback and information of HCW about the person with PLH they care for, prevention of stress and burn-out are also an important challenges to preserve institutional HCW’s QoL.

## Conclusion

This research confirms our previous results showing that caring for persons with polyhandicap have a mixed (negative and positive) impact on HCWs’ QOL. Among the determinants of their QOL, some are not modifiable such as age, perceived financial difficulties and altered health status while others are modifiable, such as coping strategies which can be improved by targeted actions.

## Data Availability

The raw data supporting the conclusions of this article will be made available by the authors, without undue reservation.

## Footnotes

^1^ https://www.cpp-sud-mediterranee-v.fr/
